# Predictors of Initiating Hormone Replacement Therapy in Postmenopausal Women: A Cross-Sectional Study

**DOI:** 10.1155/2019/1814804

**Published:** 2019-01-09

**Authors:** Hasan Çilgin

**Affiliations:** Medicine Faculty, Obstetric and Gynecology Department, Kafkas University, Kars, Turkey

## Abstract

**Objectives:**

Some of the social factors were related to hormone replacement therapy. The purpose of this study is to determine hormone replacement therapy (HRT) rates and to illustrate social factors affecting hormone replacement therapy in postmenopausal women.

**Material and Methods:**

This study comprised a total of 1052 postmenopausal women, 926 of whom were reported menopausal symptoms and sought for the treatment. 432 of these 926 participants had treated their symptoms by receiving HRT. The data was collected with a data collection form prepared by the researcher by using face-to-face interview technique. In these analyses, chi-square and Backward Logistic regression analyses were used.

**Results:**

The multivariate analyses indicated that the decision to seek treatment was influenced by a multitude of factors. These factors included location of hormone replacement therapy (OR: 12.32 [3.21-44.46] in university hospital and OR: 5.42 [2.43–13.26] in private hospital), information received about HRT (OR: 7.25 [2.14,-30.80]), physicians' counselling and involvement (OR: 5.24 [2.82-9.86]), knowledge of complications associated with HRT (OR: 6.21 [3.28-16.62]), and employment status (OR: 3.42 [1.86-5.58]). The current study identifies these factors affecting the HRT process in postmenopausal women.

**Conclusion:**

This study suggests that although the results do not demonstrate an exhaustive list of factors affecting the HRT process, they nonetheless provide evidence that the location participants applied for, physicians' counselling and involvement, participant employment status, and knowledge surrounding HRT may affect a woman's intent to receive HRT. Therefore, these results indicate that health professional influence and HRT awareness are important for HRT use. Suggestions for health care include informing women of the advantages and disadvantages of HRT to encourage popularity.

## 1. Introduction

Menopause is defined by the World Health Organization (WHO) as the complete disappearance of cyclic menstruation over a period of 12 months due to a reduction in the production of estrogen and progesterone hormones from a woman's ovaries [1]. This permanent cessation of menstrual periods can occur naturally or can be induced by surgery, chemotherapy, or radiation. Surgery, chemotherapy, and radiation can lead to estrogen deficiency and loss of reproductive function [2]. On average women experience menopause naturally around 52 years and the postmenopausal period constitutes approximately one third of women's lives. The peak frequency of vasomotor symptoms occurs about one to four years after menopause. After approximately seven to eight years from the start of menopause, symptoms typically revert to premenopausal levels. For this reason, a majority of women are under 60 years of age when vasomotor symptoms peak [3, 4].

Changes in the genitourinary system such as short-term reduction in concentration and memory, elevated coronary artery disease, cerebrovascular disease, and osteoporosis are a chronic manifestation of estrogen deficiency in the postmenopausal period [5]. Hormone replacement therapy (HRT) is a form of treatment aimed at removing the negative conditions mentioned above. HRT aims to alleviate these symptoms by replacing the reduced hormones of a postmenopausal woman, even if not at similar levels previously secreted by the ovaries in the premenopausal stage [6].

Many studies surrounding HRT reveal that a majority of women and doctors have a poor understanding of the benefits and risks of HRT. Many are unaware that HRT benefits and risks vary vastly due to numerous factors including patient's age, time since first experiencing menopausal symptoms, duration of HRT use, inclusion of progestin, and patient's medical history. Women are shown to avoid treatment of menopausal symptoms due to safety concerns surrounding HRT, potential side effect, and discomfort with discussing vaginal symptoms [7, 8].

For relief of menopause-related VMS, systemic usage of HRT with either (1) conventional estrogens/progestogens or (2) conjugated estrogens/bazedoxifene is the most effective regime. Currently, method 2 conjugated estrogens, with a selective estrogen receptor modulator such as bazedoxifene, is a very popular replacement of progestin. This method is useful for protection of the endometrium and breasts [9–11].

For women with moderate to severe menopausal symptoms who are less than 60 years old and/or women who are within 10 years of menopause and who have no contraindications such as excess risk of breast cancer or cardiovascular disease, HRT is considered an acceptable option [12–14]. Surveys surrounding postmenopause conducted on European women revealed that up to 90% declared symptoms throughout the menopausal transition, and about half found their symptoms uncomfortable. Approximately half (50–57%) of symptomatic women saw a doctor or bought herbal remedies, vitamins, or other supplements to attempt treatment. According to this study healthcare provider's communication skills and the amount of time providers spend with women seeking treatment were of utmost importance for continuation of treatment [15–17].

Many women do not possess the appropriate amount of information surrounding the current therapies for relieving physiological changes and disturbing symptoms that occur during menopause and many of these women also do not feel they have enough knowledge to make decisions about HRT [18]. Fear of adverse events causes many women to feel confused or uncertain despite recognizing that HRT can alleviate disturbing symptoms. Along with confusion or indecision about the benefits/risks of HRT, there are other obstacles that may prevent women from seeking help for menopausal symptoms. Some women feel helpless and powerless about menopause or believe that it is a natural component of aging which should be accepted [19, 20].

The purpose of this study is to determine HRT rates and to further research surrounding social factors affecting the beginning of HRT in this critically important period of life.

## 2. Materials and Methods

This face-to-face survey was conducted in department of obstetrics and gynaecology of Kafkas University, between the dates of 1st of March, 2017, and 31st of July, 2018, with 1052 postmenopausal women. In this cross-sectional study, a randomized method of sampling was selected and it aimed to reach the entire universe. All participants selected agreed to participate in the study. For standardization before data collection, apart from the author two professional doctors who would collect the data were informed and placed into 4-hour training on the aim of the study, on the nature of the questionnaire, and on conditions to be considered in data collection stage.

Nine hundred twenty-six postmenopausal women between the ages of 45-65, at the time of the survey, were eligible to complete a screening questionnaire. Patients' age ranges were equally stratified (45–49 years, 50–54 years, 55–59 years, and 60–65 years).

Patients who had a history or suspected history of breast cancer and other estrogen-based cancers, experienced early menopause, experienced prematurely active deep venous thrombosis (DVT) or a history of DVT or pulmonary embolism (PE), had a history of blood clotting disorder, and had active or a history of arterial thrombotic diseases such as myocardial infarction or stroke were excluded from the research. Women who experienced chronic liver disease or dysfunction, used herbal medicine (such as phytoestrogen) or any drugs, preferred nonpharmacological therapy, and underwent HRT treatment for less than a year were excluded. Eighty-eight percent of patients were included in the study following exclusion criteria (926/1052). The ethics review board of the hospital approved the study.

### 2.1. Variables of the Research and Collection of Research Data

The data was collected with a data collection form prepared by the researcher. Trained interviewers performed a face-to-face survey from March 2017 to July 2018 to obtain data regarding the patients' menopausal status. Obtained data from participants included the sociodemographic and biodemographic characteristics, the age at HRT initiation, disease history, reasons for starting HRT, HRT knowledge base, women's awareness of problems associated with HRT, and physician affect. The dependent variable of the study is HRT, and independent variables are the aforementioned factors.

### 2.2. Statistical Analyses

Descriptive statistical and binary logistic regression analyses were performed. Univariate and multivariate analysis was performed to analyze factors predicting outcomes. The data was analyzed in SPSS 21 (Chicago, IL, USA) packaged software. In these analyses, chi square and Backward Logistic regression analyses were used, and Odds ratio and confidence interval were calculated. The independent variables (*P*<0.05), which resulted as statistically significant in chi-square analyses, have been taken into Backward Logistic regression analyses.

### 2.3. Ethical Approval

This research was carried out at the Department of Obstetrics and Gynecology between 01.03.2017 and 01.03.2018 with the approval of the Scientific Ethics Committee of Kafkas University Faculty of Medicine (80576354-050-99/49, 01/032017). All participants were informed about the study and written informed consent was obtained from participants. The principles of the Declaration of Helsinki were followed.

## 3. Results and Discussion

### 3.1. Results

Nine hundred twenty-six female patients aged between 45 and 65 years were included in the analysis. According to this study, HRT starting rate was 46.6 percent. In a natural menopause group that ratio is 39.3% and in the surgical group it is 61.4%. When the table is monitored, there is a statistically significant relationship between the HRT rate and patient age, patient's educational and employment status, the location patients applied for, the patient's place of residence, presence of health insurance, patient's knowledge of HRT and its side effects, and physician influence. The relationship between sociodemographic features and HRT is shown in Table 1.

When considering public hospitals HRT increased by 12.3-fold in university hospital and 5.4-fold in private hospital. Considering conditions of patient employment, working in the private sector or public sector increases HRT usage by 3.4-fold. Physician guidance increases HRT use 5.2 times with reference to patients who implied that their doctor had no impact on HRT use. When considering patients who were not informed about HRT, an informed group of patients increases HRT usage by 7.2. Women aware of potential side effects associated with HRT also increased HRT usage 6.2 times in postmenopausal women (Table 2).

Univariate analysis of the potential prognostic factors affecting the iniatation of HRT found age ( OR: 1.14, 95 % CI 0.97–1.44,* p *= 0.14), women's education (OR: 1.03, 95% CI 0.93–1.22,* p *= 0.12), women's occupation (OR: 1.13, 95% CI 0.94–1.35,* p *= 0.02), residence (OR: 1.11, 95% CI 0.92–1.14,* p *= 0.13), being informed about HRT type (OR: 0.12, 95% CI 0.05–0.44,* p *= 0.03), knowing problems associated with HRT (OR: 0.14, 95% CI 0.04–0.61,* p *= 0.04), physicians' counselling and involvement (OR: 1.12, 95% CI 0.96–1.13,* p *= 0.04), the hospital they applied for (OR: 1.13, 95% CI 0.92–1.15,* p *= 0.04), and neighbourhood's effect (OR: 1.14, 95% CI 0. 98–01.13,* p *= 0.04) to be the only variables eligible for the multivariate analysis.

Among them, the hospital they applied for (OR: 12.32, 95% CI 3.21–44.46,* p *= 0.01), being informed about HRT (OR: 7.25, 95% CI 2.14–30.80,* p *= 0.03), physicians' counselling and involvement (OR: 5.24, 95% CI 2.82–9.86,* p *= 0.02), knowing problems associated with HRT (OR: 6.21, 95% CI, 3.28–16.62,* p *= 0.02) and women's occupation (OR: 3.42, 95% CI, 1.86–5.58,* p *= 0.04) were found to be an independent prognostic factor for the iniatation of HRT at the multivariate analysis.

**Figure 1:**
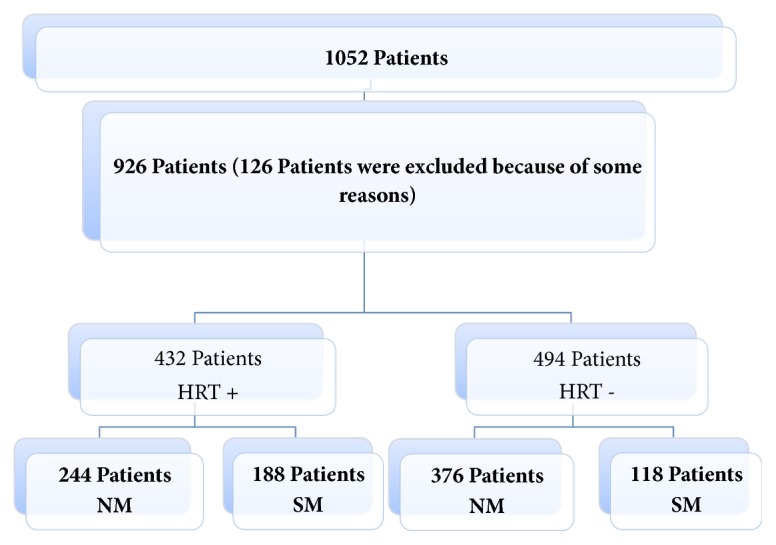
**Patient selection and total workgroup distribution scheme. NM: **natural menopause,** SM: **surgical menopause, and HRT: hormone replacement therapy.

In a total of 1052 postmenopausal women, 926 reported menopausal symptoms and sought for the treatment. 432 of these 926 participants had treated their symptoms by receiving HRT (Figure 1).

### 3.2. Discussion

Although there are a multitude of randomized, controlled studies which evaluate the factors that affect HRT usage, these studies have small sample sizes so we removed the maximum amount of information obtained from observational studies. The identification of predictive factors for the success of HRT remains an important research area that can guide clinicians and better inform patients.

In the present study, many factors affecting HRT in postmenopausal women are discussed. Patient's place of residence, age, occupation, presence of health insurance, knowledge of side effects associated with HRT, overall knowledge base surrounding HRT, spouse's job, advice and influence of neighbourhoods, the hospital they applied for, and physician's intervention have been identified as factors affecting the rate of HRT in univariate analyses performed. In multivariate analyses, the hospital they applied for, knowledge surrounding HRT, physician's influence, knowledge surrounding potential side effects of HRT, and patient's employment status have been identified as factors affecting the rate of HRT.

According to the results of this study, the use of HRT in the surgical menopause group is statistically significant. This significance can be explained in four ways: (1) presence of sudden onset of menopausal symptoms (such as vasomotor symptoms) in this group of patients, (2) physician's attitude toward HRT usage, (3) elimination of the possibility of vaginal bleeding or uterine cancer in this procedure, and (4) being a hospital-based study.

As Avis NE et al. and Politi MC et al. emphasized, vasomotor symptoms peak in frequency and frustration with symptoms peaks approximately one year after menopause. These symptoms persist at relatively high levels for approximately the following four years and typically return to premenopausal levels only at about seven to eight years after menopause and in women in our study who used HRT also predominantly in the ages between 45 and 55 years [21, 22].

Our results indicate that HRT usage increases if the patient is informed about it. In a survey conducted by Cumming GP et al., taken by 1,476 mostly perimenopausal or postmenopausal women, about half reported feeling able to make an informed choice about HRT [23].

According to the present study's results physician counselling increases HRT usage 5.2-fold compared to those patients who reported not being influenced by the doctor. Successful communication and shared decision making is a collaborative process that allows patients and medical professionals to work together and to take into account the best scientific evidence for patients. Collaborative decision-making processes also allow consideration of patient values and patient preferences for health care decisions [24].

The results indicate that patients want to be listened to, want to have facts about their diagnoses, and need to be informed about the risks and the effects of each treatment option on quality of life [25]. Women want health professionals to give honest, personalized information about their treatment options to help them choose treatment options and help them make informed decisions [26, 27].

In the study conducted, HRT was used 12.3 times more in university hospitals and 5.4 times more in private hospitals with postmenopausal women when compared to public hospitals. It is possible that HRT use increases in these settings because there are more HRT providers for menopausal symptoms in university hospitals than in private and public hospitals. Obstetrics and gynecology residency programs generally do not provide adequate menopause education and leave most residents feeling inadequately prepared to manage menopause-specific concerns [28]. Many physicians lack detailed knowledge surrounding large HRT clinical trials, and knowledge of such trials correlates moderately with prescription of HRT [29]. Recognizing that menopause is a natural process may also lead some health providers to underestimate the degree to which many women are bothered by menopausal symptoms. Studies show that doctors should speak in positive language that focuses on current, validated medical evidence including information about the benefits and risks of HRT. Doctors should also be conscious of potential prejudices and make conscious efforts to minimize the impact of these prejudices on patients [30].

The study was consistent with results of studies in the field that have found an association between patient education and HRT usage. Although women who were highly educated were more likely than the less educated women to use HRT in univariate analyses, this result was not statistically significant in multivariate analyses. This multivariate finding is not consistent with results of other studies that have found an association between education and HRT usage [31–33]. These results further indicate positive correlation between patient employment status and HRT use, which may be indirectly related to education.

Conversely, according to patient occupation, employment by the public or private sector increases the HRT rates by 3.42 times compared to patients employed outside these sectors. Unemployed patients decrease usage 0.67 times. These effects may be correlated with social and economic factors such as access to care, recognized risk for disease, control over health, confidence levels, and medical reasons. The patients who were employed may be more likely to have higher education levels and thus may be more likely to read articles about HRT and more likely to discuss personal benefits and risks with physicians as well as actively seek out this treatment.

When attempting relief of menopausal symptoms, lack of awareness and access to appropriate information are major problems that can be enhanced by providing scientifically validated information [34]. Most of the women have an incomplete understanding of current therapies relieving physiological changes and disturbing symptoms that occur during menopause. Women obtain misleading information about menopause and HRT from a variety of sources (e.g., healthcare providers, biased medical surveys, professional societies or hospitals, internet, TV, magazines, and friends and family) [27]. This study furthers the research that awareness of problems associated with HRT increases initiation by 6.2 times. Due to fear of the side effects of HRT many women feel confused, even if they realize that HRT can alleviate disturbing symptoms [12]. According to a study conducted by Nappi RE et al., roughly half of women said that HRT pills and patches could increase risk of breast cancer and stroke or blood clots; approximately one in four thought that the same local estrogen treatment was correct [35]. The EMPOWER study which was conducted on 1,858 postmenopausal 43% of women reported concern about side effects as the main reason for not using hormonal products [36]. The above results indicate that increasing awareness and knowledge surrounding HRT is essential for initiating HRT.

Although the current study's results suggest that there are no significant differences in neighbourhoods' views between groups about HRT usage, the researchers believe that dual regression analysis between neighbourhoods' effects, education, and women employment status will provide necessary and accurate information.

There are a few limitations in the present study. First, it is a hospital-based study and a selection bias exists in the recruitment of the participants. This bias may explain the dominant age group consisting of younger women and the high rate of surgical menopause in using HRT. Although the researcher excluded patients who used HRT for a period shorter than one year, not considering how many years HRT was used is another handicap of present study. This study also does not consider or assess physical health, mental health, and sexual activity.

## 4. Conclusion

According to this clinical investigation, patients' occupation, the hospital patients applied for, patients' knowledge surrounding HRT, knowledge of potential side effects associated with HRT, and physician attitude toward HRT usage are of statistical importance. The results of the current study can further research in HRT as well as increase the rates of HRT. The researchers suggest that to improve the quality of life in postmenopausal women, a higher priority needs to be placed on enhancing a menopausal woman's self-efficacy, healthcare support, and knowledge base. Enhancing these factors will likely facilitate adoption of HRT. Similarly, population-based studies with more detailed information should be conducted. Healthcare providers and physicians are required to identify the social factors affecting HRT initiation and to take measures.

## Figures and Tables

**Table 1:** Sociodemographic features of women.

Variables		**HRT(+)**			**HRT(-)**			**P**
		% (n=432)			% (n=494)			
		**NM**%	**SM**%	**P**	**NM**%	**SM**%	**P**	
		(n=244)	(n=188)		(n=376)	(n=118)		
Age	45-49	46.7	38.3	**0.95**	10.6	9.5	**0.37**	**0.017**
	50-54	28.3	33.0	**0.70**	20.0	15.7	**0.38**	**0.023**
	55-59	16.8	22.3	**0.63**	26.3	20.2	**0.41**	**0.264**
	60-65	8.2	6.4	**0.78**	43.1	54.6	**0.23**	**0.001**

Women's Education	Elementary or below	19.6	26.3	**0.65**	68.1	74.4	**0.28**	**0.024**
	Secondary school or above	80.4	73.7	**0.44**	31.9	25.6	**0.38**	**0.015**

Husband's education	Elementary or below	27.9	26.6	**0.85**	40.3	36.8	**0.84**	**0.236**
	Secondary school or above	72.1	73.4	**0.45**	59.7	63.2	**0.72**	**0.378**

Women's occupation	Unemployed	13.5	9.1	**0.53**	66.2	73.4	**0.34**	**0.020**
	Public/private sector	51.2	69.5	**0.96**	14.7	11.7	**0.25**	**0.034**
	For own	35.3	21.4	**0.45**	19.1	14.9	**0.23**	**0.275**

Husband's occupation	Employed	82.1	84.5	**0.78**	77.2	79.6	**0.32**	**0.346**
	Unemployed	17.9	15.5	**0.63**	22.8	20.4	**0.28**	**0.446**

Health insurance	Yes	86.8	83.3	**0.73**	78.6	80.4	**0.30**	**0.874**
	No	13.2	16.7	**0.96**	21.4	19.6	**0.28**	**0.675**

Residence	Village/Town	38.2	23.6	**0.46**	65.7	68.8	**0.32**	**0.034**
	City	61.8	76.4	**0.95**	34.3	31.2	**0.21**	**0.025**

Informed about HRT types	Non-informed	19.4	16.3	**0.62**	74.5	56.8	**0.23**	**0.005**
	Informed	81.6	83.7	**0.79**	25.5	43.2	**0.51**	**0.036**

Knowing problems associated with HRT	Yes	72.2	79.7	**0.84**	22.5	25.4	**0.35**	**0.021**
	No	27.8	20.3	**0.56**	77.5	74.6	**0.30**	**0.017**

Physician affects the HRT	Yes	65.2	76.8	**0.89**	20.1	23.5	**0.36**	**0.019**
	No	34.8	23.2	**0.52**	79.9	76.5	**0.29**	**0.035**

Indications	vasomotor symptoms	73.5	82.4	**0.74**				
	genitourinary syndrome	14.2	9.8	**0.69**				
	osteoporosis	12.3	7.8	**0.71**				

The hospital they applied for	University hospital	60.2	68.2	**0.95**	25.0	31.3	**0.38**	**0.032**
	Private hospital	26.1	21.2	**0.87**	18.2	25.8	**0.43**	**0.045**
	State hospital	13.7	10.6	**0.56**	56.8	42.9	**0.23**	**0.018**

Neighbourhood's effect	Yes	67.7	44.2	**0.05**	32.2	23.4	**0.22**	**0.12**
	No	22.3	55.8	**0.04**	67.8	76.6	**0.35**	**0.33**

**Table 2:** The factors affecting hormone replacement therapy.

Variables		Odds Ratio	95% CI	p
The hospital they applied for	Private hospital	5.42	2.43–13.26	**0.023**
	University hospital	12.32	3.21-44.46	**0.017**
	State hospital	1(Reference)		

Informed about HRT	Yes	7.25	2.14,-30.80	**0.015**
	No	1(Reference)		

Physician affects the HRT	Yes	5.24	2.82-9.86	**0.026**
	No	1(Reference)		

Knowing problems associated with HRT	Yes	6.21	3.28-16.62	**0.019**
	No	1(Reference)		

Women's occupation	Unemployed	0.67	0.15-3.36	**0.059**
	Public/private sector	3.42	1.86-5.58	**0.032**
	For own	1(Reference)		

## Data Availability

The data installed on SPSS 21 which were used to support the findings of this study are available from the corresponding author upon request.
